# The importance of trusting conditions for organizations’ readiness to implement mHealth to support healthy lifestyle behaviors: An interview study within Swedish child and school healthcare

**DOI:** 10.1177/20552076231181476

**Published:** 2023-06-13

**Authors:** Maria Fagerström, Marie Löf, Ulrika Müssener, Kristin Thomas

**Affiliations:** 1 Department of Health, Medicine, and Caring Sciences, 4566 Linköping University, Linkoping, Sweden; 2 Department of Biosciences and Nutrition, 27106 Karolinska Institute, Stockholm, Sweden

**Keywords:** Organizational readiness, mHealth, health promotion, child healthcare, school healthcare, implementation science

## Abstract

**Objective:**

To explore perceptions among nurses, managers, and policymakers regarding organizational readiness to implement mHealth for the promotion of healthy lifestyle behaviors in child and school healthcare.

**Methods:**

Individual semi-structured interviews with nurses (*n* = 10), managers (*n* = 10), and policymakers (*n* = 8) within child and school healthcare in Sweden. Inductive content analysis was used for data analysis.

**Results:**

Data showed that various trust-building aspects in health care organizations may contribute to readiness to implement mHealth. Several aspects were perceived to contribute trusting conditions: (a) how health-related data could be stored and managed; (b) how mHealth aligned with current organizational ways of working; (c) how implementation of mHealth was governed; and (d) camaraderie within a healthcare team to facilitate use of mHealth in practice. Poor capability to manage health-related data, as well as lack of governance of mHealth implementation were described as dealbreakers for readiness to implement mHealth in healthcare organizations.

**Conclusions:**

Healthcare professionals and policymakers perceived that trusting conditions for mHealth implementation within organizations were central for readiness. Specifically, governance of mHealth implementation and the ability to manage health-data produced by mHealth were perceived critical for readiness.

## Introduction

Health-risk behaviors still substantially contribute to the global burden of disease.^1^ About 70% of the global disease burden are caused by so-called non-communicable diseases, which are largely driven by modifiable lifestyle-related risk factors such as high blood pressure, drinking of alcohol, overweight, low fruit and vegetable intake, and sedentary lifestyles.^1^ In 2015, the United Nation member states adopted the 2030 Agenda for Sustainable Development, which states that “by 2030 reduce one third premature mortality from non-communicable disease through prevention and treatment and promote mental health and well-being”.^2^ The promotion of healthy lifestyle behaviors is particularly important among younger age groups due to the social patterning of health-risk behaviors and their long-term health impact.^3^ A population-based cohort study in Finland showed how low intakes of fruit and vegetable at age six years in low socioeconomic groups were associated with sedentary lifestyles and tobacco use during adolescence and greater risk of, e.g. hypertension and type 2 diabetes in adulthood.^3^ For the promotion of healthy lifestyles among young populations, child and school healthcare are key arenas.^4^

In the last decades, there has been an increased research focus, and growing evidence-base, for the use of interventions delivered via mobile phones (mHealth) in healthcare for the promotion of healthy behaviors. mHealth is by the World Health Organization considered to be a subcategory of eHealth (i.e. “the use of information and communications technology in support of health and health-related fields”) and have been defined as “the use of mobile wireless technologies for health”.^5^ Systematic reviews and meta-analyses indicate a small but promising effect of mHealth to promote different lifestyle behaviors among children^6,7^ and adolescents.^6–8^ For instance, a meta-analysis by He et al.^6^ aiming to determine the effectiveness of mHealth in promoting of physical activity among children and adolescents, conclude a significant improvement of the level of physical activity.

Thus, the last decades have given healthcare organizations new opportunities to use digital interventions in health promotion and disease prevention.^9^ In fact, mHealth requires fewer efforts and costs less than face-to-face interventions.^10^ However, despite promising benefits, mHealth is generally not part of ordinary work within child and school healthcare in Sweden. Adding to this, a large number of the existing studies within the mHealth research field have been effectiveness trials,^11^ and thus offer limited insights of how mHealth can be adopted by practitioners and used in everyday routines. Failing to truly implement evidence-based mHealth in healthcare, limit reach and potential health benefits among target populations.

One of the foci within implementation science is to understand barriers and facilitators for implementing innovations in healthcare.^12^ Systematic reviews^13,14^ have identified determinants for implementation on multiple levels of healthcare organizations, that all influence the extent to which healthcare professionals use mHealth in practice. Some determinants are related to *the technology* itself such as ease of use,^13,14^ evidence-base,^14^ safety,^14^ and interoperability.^13,14^ Other determinants are related to *the individual* such as familiarity with the technology^13^ and perceived benefits.^14^ Determinants on *organizational levels* are also highly prevalent including culture,^14^ workflow-related factors,^14^ training,^13,14^ access to guidelines,^14^ and resources.^13,14^ However, previous research has mainly investigated implementation in terms of the relationship between users of technology (e.g. health care professionals and patients) and the technology.

An organization's readiness for change (rather than practitioners per se) has been proposed to be critical for successful implementation.^15,16^ According to the organizational readiness for change theory by Weiner et al.^15^ readiness to implement change is the extent to which individuals within an organization are psychologically and behaviorally prepared to implement a change. It has been argued that half of all implementation efforts fail due to poor organizational readiness for change.^16^ The readiness to implement digital interventions in healthcare organizations has been called “eHealth readiness”.^17,18^ Previous research indicates that eHealth readiness may include several aspects of readiness, such as technological (the ability to meet technology requirements), motivational (perceived benefits), engagement (engagement of stakeholders at all levels), and acceptance at the individual level.^17^ However, research on eHealth readiness covers the implementation of a broad and diverse group of innovations that often target healthcare professionals, such as healthcare records and clinical information systems.^19^ mHealth, on the other hand, primarily includes interventions that ultimately aim to reach out to the patient. This means that within healthcare, readiness to implement mHealth requires not only readiness to incorporate the new technology, but also readiness to integrate the intervention into routine visits and to engage end-users. Consequently, organizational readiness to implement mHealth might differ from readiness to implement eHealth. More research is thus needed that investigates readiness to implement mHealth tools specifically, rather than eHealth in general.

## Methods

### Study aim

To explore perceptions among nurses, managers, and policymakers regarding organizational readiness to implement mHealth for the promotion of healthy lifestyle behaviors in child and school healthcare.

### Study design

An explorative study including semi-structured interviews^20^ with nurses (*n*  =  10), managers (*n*  =  10), and policymakers (*n*  =  8) in child and school healthcare. Inductive content analysis following Elo and Kyngäs^21^ was used in the data analysis. The Consolidated Criteria for Reporting Qualitative Research^22^ checklist was used as a guide in the reporting of the study (Supplemental Appendix 1).

### Study setting

The research was conducted in child and school healthcare settings in three regions within Sweden. These regions include urban and rural settings as well as high and low socioeconomic areas. Both child and school healthcare are commissioned to work with health promotion and disease prevention, including offering regular health visits. Health visits cover aspects such as health-risk behaviors and general well-being. Registered nurses specialized in public health, children's health or school health typically lead this work. Organizationally, child healthcare centers are a part of primary care.^4^ The school principal is responsible for school healthcare, and healthcare professionals (e.g. nurses, psychologists) can be employed centrally, outsourced to private contractors, or employed in-house by the schools. Both child healthcare and school healthcare are governed by Swedish health and medical law, although in practice they work separate from the wider healthcare organization.

### Recruitment and participants

A purposeful sampling was employed considering location, organization size, socioeconomic area (child healthcare), and educational orientation (school healthcare). Breadth and information-richness was further sought by the recruitment of nurses and managers within two different randomized controlled trials: the MINISTOP 2.0 trial in child healthcare^23^ and the Life4YOUth trial^24^ within school healthcare, which ensured that informants were experienced in using mHealth. MINISTOP 2.0^23^ is an app promoting healthy diets and physical activity among families with 2–3-year-old children. Life4YOUth^24^ is an app that promotes healthy diet, physical activity, smoking cessation, and non-risky drinking among adolescents (16–18 years). Eligible individuals received information about the study and an invitation to take part via email. No other relationship was established between the interviewers and informants prior to the study. Please see Table 1 for the characteristics of the informants.

**Table 1.** Number and characteristics of informants in terms of gender, years of age and years in profession.

	Nurses (*n*)		Managers (*n*)		Policymakers (*n*)	
	Child healthcare	School healthcare	Child healthcare	School healthcare	Child healthcare	School healthcare
Gender						
Male	-	-	3	-	4	4
Female	6	4	2	5	-	-
Years of age						
≤30	-	-	-	-	-	-
31–40	3	-	2	-	-	-
41–50	3	2	-	-	3	2
≥51	-	2	3	5	1	2
Years in profession						
≤3		-	2	2	-	-
4–6	2	-	1	2	1	1
7–9	2	1	1	1	-	1
≥10	2	3	1	-	3	2

*Nurses:* Inclusion criteria were to be currently working at a child or school healthcare center participating in MINISTOP 2.0^23^ or Life4YOUth^24^ trials, and to have experience of using mHealth in work routines. The exclusion criterion was not using mHealth during routine health visits. Seven nurses involved in school healthcare were invited to take part. Three did not respond, giving a total of four school healthcare nurses being interviewed. All nurses at the child healthcare centers participating in the MINISTOP 2.0 trial^23^ were invited to take part (*n*  =  62). The first six nurses who responded to the invitation represented heterogeneous contexts and were therefore interviewed. In total, ten nurses were interviewed. None had experience of using mHealth before participating in the MINISTOP^23^ or Life4YOUth^24^ trials. Child healthcare nurses represented small (*n*  =  3), medium (*n*  =  2), and large (*n*  =  1) organizations and were located in low (*n*  =  1), medium (*n*  =  2), and high (*n*  =  3) socioeconomic areas. School nurses represented small (*n*  =  2), medium (*n*  =  1), and large (*n*  =  1) schools, including theoretical, vocational, and introductory courses.

*Managers:* The inclusion criterion was a current management position at a child or school healthcare center participating in MINISTOP 2.0^23^ or Life4YOUth^24^ trials. In total, 24 individuals were invited to take part; two declined and 12 did not respond, resulting in 10 managers being interviewed. None of the informants had experience of implementing mHealth for health promotion or disease prevention purposes, although managers within child healthcare did have experience of implementing other types of mHealth. All the managers within child healthcare were operational managers, representing small (*n*  =  3), medium (*n*  =  1), and large (*n*  =  1) institutions, located in low (*n*  =  2), medium (*n*  =  2), and high (*n*  =  1) socioeconomic areas. Managers within school healthcare worked in positions such as coordinating nurses or as operations managers with varied sizes of school healthcare organizations depending on their management assignment.

*Policymakers:* The inclusion criterion was to be responsible for or involved in organizational eHealth strategy. A total of 19 individuals were invited to take part; three declined and eight did not respond, resulting in a total of eight informants from eight different healthcare organizations. All these informants had a strategic responsibility at regional, municipal or organizational levels and had experience of taking decisions regarding mHealth implementation. Informants within school healthcare functioned as policymakers for both the educational and school health arena, and all had experience of implementing digital tools within teaching.

### Data collection

Three semi-structured interview guides were developed, one each for the nurses, managers, and policymakers (Supplemental Appendix 2). All three guides included questions about experiences of implementing mHealth and perceptions of what is needed within the organization to be prepared to implement mHealth. The guides were pilot tested and tailored to suit each informant group in terms of wording and minor content adaptations. The interviews were conducted via telephone between April and October 2021 by a female PhD student and physiotherapist (MF). However, the pilot interview was carried out by a female researcher experienced in qualitative methods (KT). Only the interviewer and the informant were present during the interviews. An ongoing discussion regarding interview technique and content was held between MF and KT. A total of 28 interviews were conducted, each one lasting between 21 and 57 min. The main interviewer (MF) had no previous experience of the research area or research setting. The last author, KT (behavioral scientist and female researcher) and co-author UM (occupational therapist and female researcher) were experienced in both the research field and setting. Before the first interview, a “pre-understanding” was written by MF. Directly after each interview field notes were generated by MF. These contained thoughts and reflections regarding the interview in terms of its content and process. Field notes were thus predominantly used as a tool in the initial part of analysis. Each interview started by providing information about the study and the interviewer (physiotherapist, PhD student within implementation science with no association with the mHealth trials^23,24^), and gaining verbal informed consent. Probes and clarifying questions were added as needed during each interview. The interviews were audio-recorded and transcribed verbatim. After 28 interviews, the material was assessed as having captured the study aims to a sufficient extent.

### Data analysis

The data was analyzed using inductive content analysis,^21^ involving three authors (MF, KT, UM). Both manifest and latent content were analyzed, and the unit of analysis was individual organizational members (nurses, managers and policymakers). Firstly, the data was read repeatedly by MF and KT to obtain a sense of the whole and to identify words or phrases describing readiness to implement mHealth. These words and phrases were condensed to meaning units, which were thereafter assigned an open code relating to the study aims. MF and KT performed the open coding independently, using NVivo 20 to assist data administration. Through a process of abstraction, the codes were then further condensed and divided into sub-categories, categories, and a main category. MF and KT performed the abstraction process independently, and the emerging categories were repeatedly discussed between MF, KT, and UM until consensus was reached. Analysis notes were taken throughout by MF.

## Results

**Figure 1.**
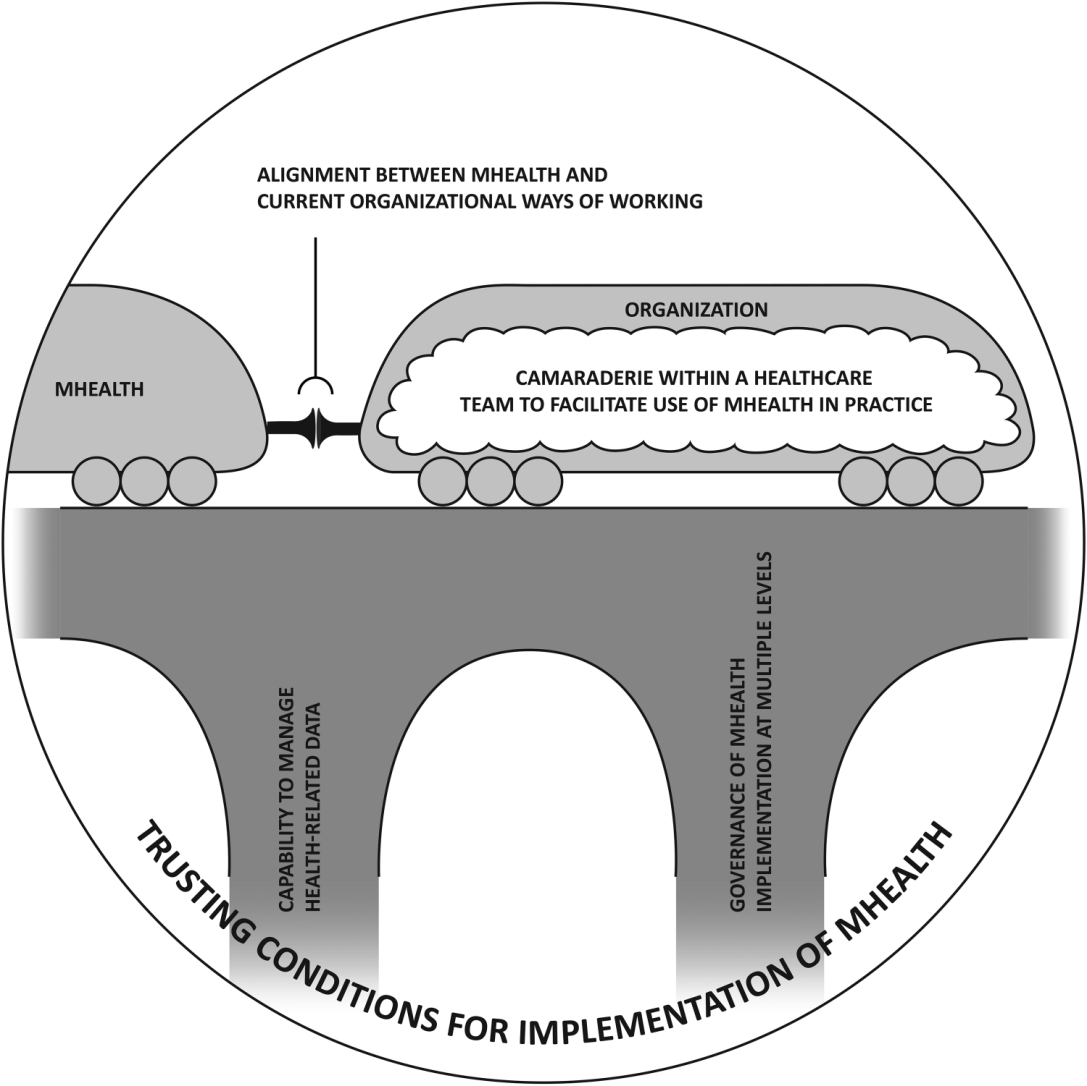
Illustration of main findings. Several aspects (i.e. categories) were found to contribute to trusting conditions for implementation of mHealth (main category): (a) capability to manage health-related data; (b) governance of mHealth implementation at multiple levels; (c) alignment between mHealth and current organizational ways of working; and (d) camaraderie within a healthcare to facilitate use of mHealth in practice. Poor capability to manage health-related data and lack of governance were perceived to act as dealbreakers and are therefore illustrated as cornerstones for trusting conditions for implementation of mHealth.

According to data, various trust-building aspects in an organization contribute to its readiness to implement mHealth (Table 2). Several aspects were perceived by informants as contributing to the main category, *trusting conditions for implementation of mHealth*: (a) how health-related data can be stored and managed; (b) how mHealth align with current ways of working in an organization; (c) how implementation of mHealth is governed; and (d) camaraderie within healthcare teams to facilitate use of mHealth in practice (Table 2). Figure 1 illustrates the main findings (main category and categories). Translated quotes have been included throughout the results section to illustrate and exemplify.

**Table 2.** Overview of main category, categories, and sub-categories.

**Main category** Trusting conditions for implementation of mHealth	
Categories	Sub-categories
Capability to manage health-related data	Interoperability between data management systems Capacity within data management systems
	Legal and ethical frameworks governing management of health-related data
Alignment between mHealth and current organizational ways of working	Adapt mHealth content to organizational goals, clinical routines and values Accustom organizational culture to digital working methods
Governance of mHealth implementation at multiple levels	Clarify the role of mHealth in healthcare Coordinate implementation of mHealth in healthcare Ensure quality of available mHealth interventions Top-down management at clinical level
Camaraderie within a healthcare team to facilitate use of mHealth in practice	Collaborative approach Opportunities for peer-learning about mHealth Nurturing of respectful and trustworthy relationships

### Trusting conditions for implementation of mHealth

The data showed that various trust-building aspects in an organization contribute to its readiness to implement mHealth interventions in practice. These were both structural, such as governance and management of health-related data, as well as related to process of care such as the importance of camaraderie within healthcare teams.

Specifically, trusting conditions regarding management of health-related data, concerned trust in the organization's capability to manage the data that was produced by mHealth, in terms of data security, capacity and interoperability. Alignment between mHealth and current ways of working within an organization was described by informants as promoting trust in mHealth and thus building readiness. The governance of mHealth implementation at multiple levels within and outside of the organization were perceived as promoting trusting conditions by offering guidance to organizations. Trusting conditions were also talked about in terms of trust in colleagues and managers, which in turn promoted consensus, collaboration, and peer-learning. Finally, trust in one's own ability to use mHealth was considered crucial for being ready to use mHealth in routine practice and recommending it to end-users.

*We would have to ensure that it [the data] won’t somehow end up wrong, because this is usually quite sensitive information, so I would probably be very careful… spontaneously, the difficulties exceed the possibilities.*

Policymaker, school healthcare, #7

*Capability to manage health-related data.* Poor capability to manage health-related data produced by mHealth was described as a dealbreaker for readiness and successful implementation. Informants stressed that unless there was an appropriate structure put in place for data management, the organization would not be ready for mHealth at all, even though other aspects of organizational readiness were present. In addition, legal and ethical requirements for management of data were expressed as important for readiness as mHealth often need to collect and produce private and sensitive data. Informants perceived the laws governing mHealth to be complex and difficult to interpret. Furthermore, existing laws governing mHealth were perceived to be outdated, and primarily based on medical technology products, and thus offered limited guidance for most mHealth interventions used for health promotion purposes. Consequently, informants described the laws governing mHealth to be difficult to understand and use, which was perceived to compromise organizations’ readiness to implement mHealth.

Furthermore, informants stressed the importance of having capacity within the local data management system and interoperability in terms of combining and sharing data, both within the data management system and in relation to electronic health records. Informants perceived current system interoperability to be poor, which contributed to compromised readiness to implement mHealth. In contrast, mHealth that were designed not to collect health data bypassed this need for data storage and management. For example, informants perceived organizations to be more ready to implement mHealth if they predominantly offered tips and advice, rather than collecting or monitoring health data.

*This requires integration, and we dońt have the IT [information technology] architecture in place. Because, the problem is, if you purchase an app, or build an app, it operationalizes completely by itself in a vacuum, but organizationally it is not in a vacuum.*

Policymaker, child healthcare, #22

*Alignment between mHealth and current organizational ways of working*. How well mHealth aligned with current organizational ways of working were also perceived to contribute to readiness to implement mHealth. Current ways of working were described in terms of goals, clinical routines, values as well as culture within the organization. Alignment was talked about as the degree of compatibility between the *mHealth content* and current organizational ways of working, but also in terms of compatibility between current ways of working and *mHealth as a new digital method* to promote healthy lifestyle behaviors.

Organizational goals were described as context-specific and closely linked to the purpose of the organization. In the case of child and school healthcare, key goals for practice were to work with health promotion and disease prevention using a holistic approach to health. Aligning the mHealth content with organizational goal was believed to enhance readiness. Furthermore, informants perceived that readiness was enhanced if the mHealth content aligned with clinical routines. For example, readiness to integrate technology into routine visits within these settings was perceived to be increased if mHealth content targeted topics typically covered during routine visits. Informants emphasized the need for child and school healthcare to work according to common values within the organization, such as providing equal care and working in an evidence-based manner. Informants stated that knowing that a mHealth intervention was evidence-based, easily accessible, and tailored to specific end-user groups would be in line with current ways of working and thus promote readiness. However, informants also expressed current ways of working in terms of a culture within the organization. Culture concerned informants’ perceptions of professional roles and identities, and professionals were described as having to become accustomed to using mHealth as a *new method* in general*.* To become accustomed was a slow habituation process and parts of the culture were considered difficult to align with mHealth at all. For example, there was a concern that mHealth disempowered trusting relationships between nurses and end-users, including nurses’ sense of control. Also, mHealth's fast-changing digital landscape was described as contrasting with the traditional way of working in healthcare, making it difficult to keep up with and adapt to aspects such as commercially available mHealth, or to know which mHealth could be trusted to recommend to patients.

*It [the mHealth] is easy to use during the routine visits, because we talk about so many parts that the app contains, so you can easily connect… listen here, we have talked about food, physical activity and screen time; look here, let me show you [the mHealth]…*

Nurse, child healthcare, #1

*Governance of mHealth implementation at multiple levels.* Governance of mHealth implementation was described as guidance, coordination, and strategies for how organizations were to work with mHealth. Informants expressed a belief that governance could promote readiness to implement mHealth in healthcare organizations by clarifying the purpose and role of mHealth in healthcare practices. Lack of governance of mHealth implementation was described as a dealbreaker for readiness and successful implementation.

Informants highlighted a need for both national and regional guidance regarding mHealth use in healthcare in general. Guidance was perceived to be needed to elucidate the role that mHealth should have in healthcare practice and to coordinate stakeholders at both regional and national levels. Informants described that the system currently lacked guidance which was thought to compromise the readiness of health care organizations to truly implement mHealth. Specifically, informants conveyed the need for an explicit national strategy to ensure the quality of the large number of available mHealth. It was perceived that such a national strategy would promote readiness by facilitating initiatives and the use of mHealth in practice. Professionals described being left alone to find suitable mHealth, and to draw their own conclusions about interventions’ quality, safety, and cost-effectiveness. Furthermore, at a clinical level, informants talked about governance in terms of that readiness to implement mHealth could be promoted through top-down guidance by “the right leader.” However, who the “right leader” was, was context-dependent and could consist of knowledge centers or operational managers.

*Just as we have national guidelines for different care processes, I would like that, within this field, we had nationally found that these three apps meet the requirements for evidence-based guidelines and are in line with knowledge requirements and all that stuff, so they get a tag or something. Then it would be much easier for the professionals to recommend apps that are approved.*

Policymaker, child healthcare, #17

*Camaraderie within a healthcare team to facilitate use of mHealth in practice.* Camaraderie was described as respectful and trustworthy relationships among colleagues within a healthcare team. Camaraderie was perceived to be important for readiness by creating an open climate where both hopes and fears about mHealth could be expressed and improving opportunities for learning about mHealth.

A collaborative approach within a team facilitated building new routines. For example, informants talked about the need for new actions to create “end-user readiness,” i.e. making end-users aware of the mHealth before they were introduced to it during health visits, for example advertisements for mHealth. Camaraderie promoted informal peer-learning whereby team members exchanged experiences, shared insights, and asked questions, enabling the team to practice mHealth together in a nonjudgmental way. This was highlighted as an important strategy for enhancing nurses’ competence and confidence, which was considered crucial for nurses’ propensity to promote mHealth. In addition, camaraderie within the healthcare team could also help in the management of different characteristics and personalities among professionals, such as resistance to change. The team was considered to include co-workers with similar roles and responsibilities (e.g. nurse colleagues) but might also involve organizational members who worked indirectly with implementation efforts, such as managers and mentors.

*People who are more used to working with it [the mHealth] support the ones who aren’t so used to it, it's an openness to come to the door and ask, so we become some kind of intermediary before going to the best expertise, helping each other.*

Manager, school healthcare, #19

## Discussion

Our findings suggest that various trust-building aspects need to be present in order for a healthcare organization such as child and school healthcare to be ready to implement mHealth. Aspects that contribute to trusting conditions included capability to manage health-related data as well as governance for mHealth implementation at national, regional, and clinic levels. In addition, the findings highlight the importance of aligning new technology with current organizational ways of working, as well as promoting camaraderie within healthcare teams.

The findings include both intra- and interpersonal aspects of trust in the human–technology–organization interaction. Earlier research has established that trust between team members and leadership contributes to readiness for change and implementation.^25^ The importance of team dynamics has also been emphasized in mHealth research^14^; however, less so in the eHealth readiness literature.^17^ Readiness to implement mHealth may be more dependent upon relationships within the healthcare team because the ultimate goal is to reach end-users (e.g. patients and clients). mHealth implementation requires end-user trust,^26^ which in turn is influenced by the attitudes and behaviors of professionals.^27^ Our data indicate the importance of peer-learning and self-efficacy within healthcare teams to ensure readiness to use mHealth in practice. Thus, improved digital competence among staff may be a way to align organizational culture with digitalization and mHealth. As pointed out by Greenhalgh et al.^28^ acceptance by the involved professionals is the key to whether new technology succeeds or fails at a local level. Furthermore, to clarify the difference between eHealth readiness and organizational readiness to implement mHealth, we suggest that the term “mHealth readiness” could be used.

In this study, we conclude that poor capability to manage health-related data can be a dealbreaker for readiness. Lack of interoperability is a well-documented barrier to mHealth implementation^13,14^ and eHealth readiness^17^ and thus needs to be considered during mHealth development and implementation. However, mHealth that do not collect health data bypass the need for data storage and management and is thus more ready to be implemented. Additionally, readiness could be enhanced by clarifying how each tool meets safety requirements.^29^

Leadership and governance could be particularly important for the implementation of mHealth because the digitalization of care implies a new paradigm for healthcare organizations. Informants perceived national steering to be poor despite the Swedish government's vision for the country to be leading in the world at taking advantage of digitalization and eHealth by 2025,^30^ with major investments being made. Research from the United Kingdom has concluded that the absence of national steering is one of the main barriers for eHealth implementation in healthcare.^31^ In addition, even though county councils may have regional guidelines for how to handle mHealth tools, Sweden lacks instances of “quality control” for mHealth that are not classified as a medical devices.^32^ Informants argued that this led to professionals being left alone to draw their own conclusions about specific mHealth, which compromised organizational readiness.

The findings of this study suggest that new technology needs to fit with existing organizational values and visions, a premise that is not new to implementation science. Both Rogers^33^ and Greenhalgh et al.^27^ have proposed the need for compatibility of an innovation, or an “innovation-system fit,” meaning that an innovation which fits existing values, norms, goals, skills, and supporting technologies is more likely to be implemented successfully. Yet, informants stated that alignment between mHealth and existing values and visions is often overlooked. It is possible that rapid development and a willingness to follow the digital line of end-user society make us “speed-blind”. One way to tackle this issue could be to tailor mHealth against specific organizational culture and ethos by involving professionals during the design and development of a mHealth. Another way could be to use approaches such as implementation mapping, i.e. to considering implementation aspects already during the mHealth development phase.^34^ However, our findings also underline the need for mHealth governance that clarifies the role of mHealth in routine care.

The findings can be partly understood and explained using Weiner's Theory of Organizational Readiness for Change (ORC).^15^ For example, our findings indicate that contextual factors contribute to the willingness (such as a desire to follow end-user needs) and ability (such as the importance of data management) to implement mHealth. Furthermore, in line with ORC,^15^ our findings highlight that the contextual factors also reside outside of an organization. In our data, this was illustrated by the importance of governmental steering.

However, our findings also indicate that ORC may not be able to capture all the nuances of readiness to implement mHealth. Indeed, the findings indicate that when it comes to implementing mHealth, there may be dealbreakers (poor capability to manage health-related data, lack of mHealth governance) meaning that high readiness in terms of, e.g. camaraderie may not compensate for poor data management. Also, camaraderie within health care teams may facilitate implementation per se, whereas appropriate data storage and management facilitate mHealth implementation specifically. Consequently, to understand and promote readiness to implement mHealth in healthcare, intervention-specific facets of organizational readiness are needed. Such a heuristic is proposed by Scaccia and coworkers.^35^ They suggest that organizational readiness involves the motivation to implement an innovation and, furthermore, separates general capacity from innovation-specific capacity within an organization. In addition, they propose that, if one of these factors is close to zero, high degrees of the other will not accomplish readiness.

### Methodological considerations

Some limitations and methodological issues should be considered when interpreting our findings. The organizations in this study were positioned within Swedish child and school healthcare, which primarily take a health promotional holistic approach, and work rather independently on the fringes of other parts of Swedish healthcare. Furthermore, the study primarily concerned mHealth for health promotion and disease prevention purposes, and the findings may thus have limited transferability to other types of mHealth in other areas of healthcare. A methodological strength is that the informants were recruited from organizations participating in mHealth trials,^23,24^ which ensured that they all had mHealth experience. On the other hand, organizations voluntarily enrolling onto mHealth trials may be more open to change. In addition, a relatively significant number of potential informants declined to participate in an interview, and there is a risk that organizational members who agreed to participate in interviews had a more positive attitude to mHealth. The majority of informants within this study were women. All nurses participating in interviews were women, however, male child and school healthcare nurses in Sweden are rare. The sample can therefore be considered representative for child and school healthcare nurses in general. Within the policymaker group, however, all informants were men. Including women within this group would have made the sample more representative of the Swedish healthcare system and thus increased the transferability of the study findings.

The trustworthiness of the findings was considered in several ways. Purposeful sampling and heterogeneity endorsed credibility by allowing the collection of rich material.^36^ Telephone interviews were conducted to support heterogeneity in terms of geographic location, but at the same time support the feasibility of the study. Earlier research has shown minimal disparities in quality of data that are collected via telephone compared to face-to-face interviews.^37^ Heterogeneity was further enhanced by the informants’ experiences of two similar but different apps, and two similar but different settings. However, including perspectives from other informant groups could have further enriched the data, for example, quality improvement officers. Transferability was considered by means of the detailed reporting of aspects such as sampling and research setting.^38^ Credibility was further endorsed by pilot testing the interview guides,^39^ probing during interviews,^38^ and investigator triangulation.^39^ Dependability was mainly addressed by following the steps laid out by Elo and Kyngäs,^21^ but also through taking field notes during data collection. Dependability and confirmability were further enhanced by the keeping of an audit trail.^39^ In addition, confirmability was improved, besides discussions between the researchers, by setting out a written preunderstanding (MF) which clarified any expectations and beliefs that might influence data collection and analysis.^36^

## Conclusions

Policymakers and professionals perceived an organization's readiness to implement mHealth in terms of trusting conditions for implementation of mHealth. Poor capability to manage health-related data produced by mHealth, as well as lack of governance of implementation, may be dealbreakers for readiness and should be carefully considered prior to implementation of mHealth in healthcare. Our findings cannot fully be explained by existing organizational readiness to change theory but highlights a need to also include innovation-specific components in theory development.

## Supplemental Material

sj-docx-1-dhj-10.1177_20552076231181476 - Supplemental material for The importance of trusting conditions for organizations’ readiness to implement mHealth to support healthy lifestyle behaviors: An interview study within Swedish child and school healthcare Click here for additional data file. Supplemental material, sj-docx-1-dhj-10.1177_20552076231181476 for The importance of trusting conditions for organizations’ readiness to implement mHealth to support healthy lifestyle behaviors: An interview study within Swedish child and school healthcare by Maria Fagerström, Marie Löf, Ulrika Müssener and Kristin Thomas in DIGITAL HEALTH

sj-docx-2-dhj-10.1177_20552076231181476 - Supplemental material for The importance of trusting conditions for organizations’ readiness to implement mHealth to support healthy lifestyle behaviors: An interview study within Swedish child and school healthcare Click here for additional data file. Supplemental material, sj-docx-2-dhj-10.1177_20552076231181476 for The importance of trusting conditions for organizations’ readiness to implement mHealth to support healthy lifestyle behaviors: An interview study within Swedish child and school healthcare by Maria Fagerström, Marie Löf, Ulrika Müssener and Kristin Thomas in DIGITAL HEALTH

## Abbreviations

eHealth: the use of information and communications technology in support of health and health-related fields
mHealth: the use of mobile wireless technologies for health
ORC: the theory of Organizational Readiness for Change.
