# Supplementary material for: The importance of trusting conditions for organizations’ readiness to implement mHealth to support healthy lifestyle behaviors: An interview study within Swedish child and school healthcare
Source: Digit Health. 2023 Jun 13;9:20552076231181476. doi: 10.1177/20552076231181476 (PMC10286530; doi:10.1177/20552076231181476)
Supplement: sj-docx-2-dhj-10.1177_20552076231181476 - Supplemental material for The importance of trusting conditions for organizations’ readiness to implement mHealth to support healthy lifestyle behaviors: An interview study within Swedish child and school healthcare [file sj-docx-2-dhj-10.1177_20552076231181476.docx]

**Nurses**

Can you tell me about your professional role and work?

How does your organization currently work with mHealth?

How do you perceive the need for mHealth in child/school healthcare?

What has motivated you and your colleagues to participate in the Life4YOUth/MINISTOP trials?

How have you and your colleagues proceeded to use the Life4YOUth/MINISTOP apps in your daily work?

Can you tell me about the feeling within the group during the Life4YOUth/MINISTOP trials?

What would you and your colleagues need to enable you to implement mHealth, such as Life4YOUth/MINISTOP?

Please share your thoughts about whether or not [name of the organization] has what is required to implement mHealth.

Please share your thoughts if there are any special considerations prior implementing mHealth, compared to other digital interventions.

Please share your thoughts about the future and the use of mHealth, such as Life4YOUth/MINISTOP.

**Managers**

Can you tell me about your professional role and work?

How does your organization currently work with mHealth?

How do you perceive the need for mHealth in child/school healthcare?

How do you perceive your and the child/school healthcare nurses’ motivation to implement mHealth?

How have you and the child/school healthcare nurses proceeded to use the Life4YOUth/MINISTOP apps in the daily work?

How do you view your role as a manager, when implementing new routines?

Can you tell me about the feeling within the group during the Life4YOUth/MINISTOP trials?

Please share your thoughts about whether or not [name of the organization] has what is required to implement mHealth.

Please share your thoughts if there are any special considerations prior implementing mHealth, compared to other digital interventions.

Please share your thoughts about the future and the use of mHealth, such as Life4YOUth/MINISTOP.

**Policymakers**

Can you tell me about your professional role and work?

What does mHealth mean to you?

Why was the decision to implement mHealth made?

How do you perceive the need for mHealth in child/school healthcare?

Please to tell me about some mHealth features that currently are used in [name of the organization].

Imagine child/school healthcare were to implement mHealth; please share your views on [name of the organization] insights into what is required to implement.

Please share your thoughts about whether or not [name of the organization] has what is required to implement mHealth.

Please share your thoughts if there are any special considerations prior implementing mHealth, compared to other digital interventions.
